# Epidermal inclusion cyst after breast reduction mammoplasty

**DOI:** 10.1016/j.radcr.2026.02.056

**Published:** 2026-03-22

**Authors:** Ashley Park, Valerie Lugo-Rodriguez, Katerina Freyre Diaz

**Affiliations:** Department of Radiology, Maimonides Medical Center, 4802 10th ave, Brooklyn, NY 11219, USA

**Keywords:** Benign breast lesions, Reduction mammoplasty, Epidermal inclusion cyst

## Abstract

We report a case of a 74-year-old female with left breast multicentric invasive ductal carcinoma status post left mastectomy with reconstruction and right breast symmetrizing reduction mammoplasty presenting with a new asymmetry of the right breast that was found to be an epidermal inclusion cyst (EIC) upon core needle biopsy. The aim of the case report is to characterize the pathogenesis of EIC as well as its clinical and imaging features. This benign entity is of significance due to its malignant potential to squamous cell carcinoma.

## Introduction

An epidermal inclusion cyst (EIC) is a common entity that can appear anywhere in the body where the epidermal elements proliferate and implant within the underlying dermal layer. The accumulation of epithelial and keratinous debris results in a lamellated keratin-filled cyst. However, the EIC of the breast is a rare, benign entity that have the potential to be malignant [1]. In this case report, we describe a case of EIC status post reduction mammoplasty and provide clinicoradiological and cytohistological insight, which has been seldomly reported in the literature.

## Case report

The patient is a 74-year-old female with a history of left breast multicentric invasive ductal carcinoma, status post left mastectomy with reconstruction and right breast symmetrizing reduction mammoplasty, presenting for follow-up imaging of the right breast. Her past medical history includes hypertension, hyperlipidemia, and diabetes mellitus. The initial diagnosis of left breast cancer was made in November 2022, when she presented with left nipple retraction, a palpable left breast mass, and left upper extremity swelling. She underwent left total mastectomy and axillary lymph node dissection, as well as immediate reconstruction and right reduction mammoplasty (February 2023).

Pathology revealed T2(2)N1 well-differentiated, estrogen receptor (ER)-positive/progesterone receptor (PR)-positive/human epidermal growth factor receptor 2 (HER2)-negative invasive ductal carcinoma with associated intermediate nuclear grade ductal carcinoma in situ (DCIS), cribriform pattern, with necrosis in the left breast, and a positive intramammary lymph node in the central region with up to a 0.3 cm metastatic deposit by direct extension. She subsequently completed curative-intent radiation therapy (June 2023) and began anastrozole therapy (December 2023).

An exchange of the left breast tissue expander and insertion of an implant were performed in September 2024. Follow-up MRI showed a 12 cm fluid collection in the left breast within the mastectomy bed without suspicious enhancement in either breast. A nuclear medicine bone scan of the whole body was negative for metastasis.

In October 2024, the patient underwent removal of the previous implant and debridement of the skin, subcutaneous tissue, and granulation tissue in the left breast due to infection. Surgical pathology demonstrated invasive ductal carcinoma involving the dermis and subcutaneous tissue, indicating recurrence of left breast cancer. Subsequently, left wide local excision with extensive sampling was performed that showed no residual invasive or in situ carcinoma.

**Fig. 1:**
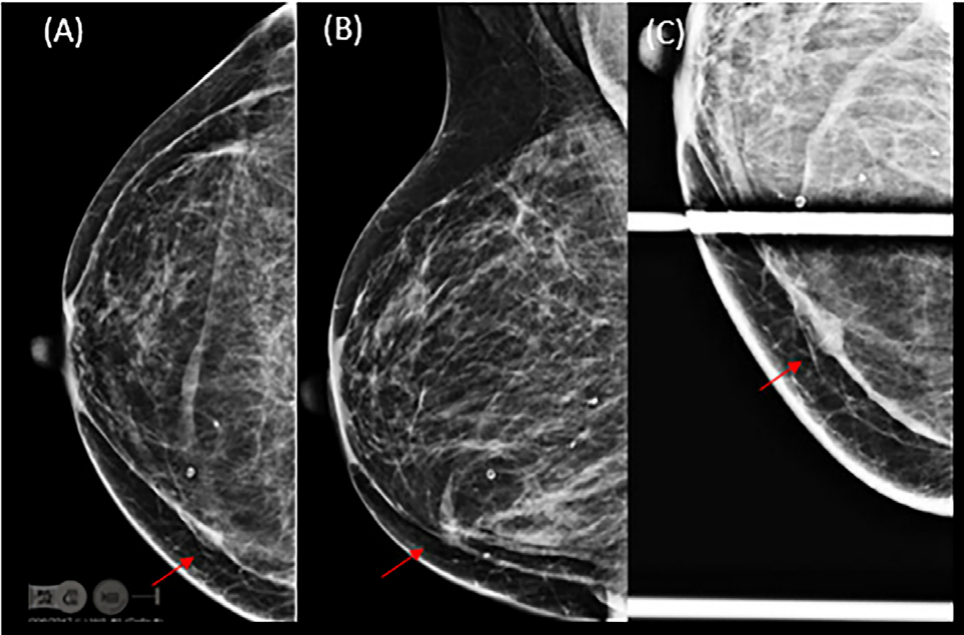
(A) Right cranio-caudal, (B) medio-lateral oblique and (C) cranio-caudal spot compression views show reduction mammoplasty post-surgical changes and an asymmetry in the inner breast.

**Fig. 2:**
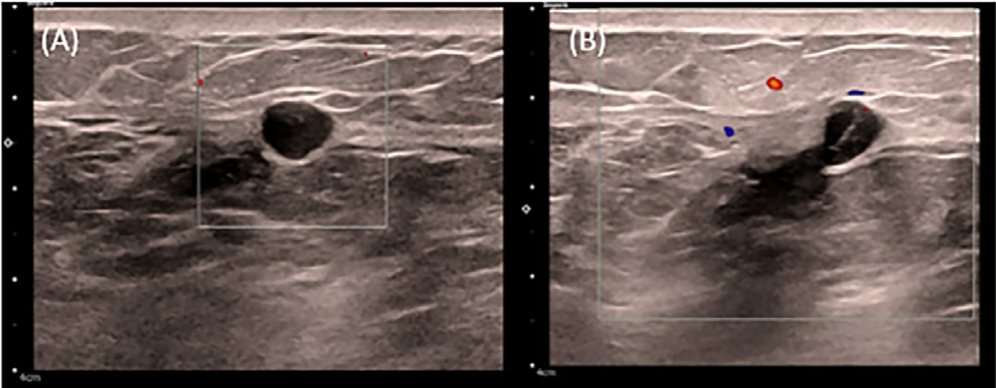
(A) Right breast targeted sonogram with color doppler shows a round, circumscribed, hypoechoic mass at 3:00 axis, 7 cm from the nipple measuring 0.7 × 0.6 × 0.8 cm. (B) Continuous with this finding, there is an elongated, irregular, hypoechoic mass measuring 1.4 × 0.6 × 1.4 cm.

**Fig. 3:**
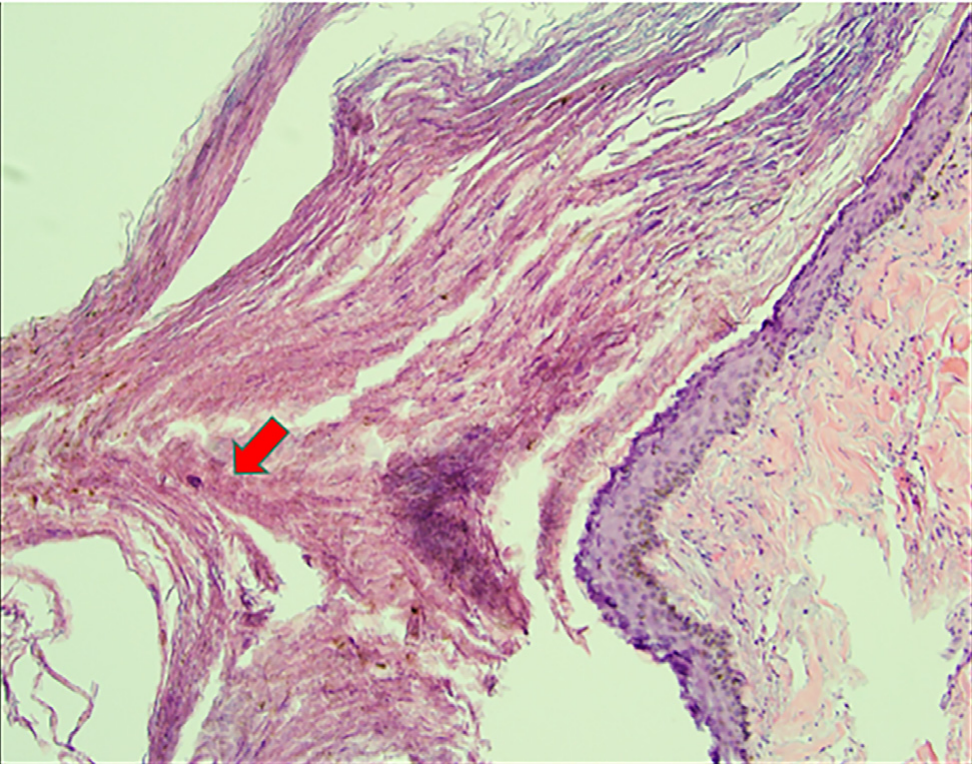
Core needle biopsy of the new right breast asymmetry shows a cystic structure lined by squamous epithelium containing laminated keratinous material, indicating epidermal inclusion cyst.

Follow-up digital mammography with tomosynthesis showed scattered fibroglandular densities and postsurgical changes due to reduction mammoplasty, with a new asymmetry on the craniocaudal (CC) view in the inner right breast (Fig. 1). Targeted ultrasound of the asymmetry showed a round, circumscribed, hypoechoic mass at 3:00, 7 cm from the nipple, measuring 0.7 × 0.6 × 0.8 cm. Contiguous with this finding, at 3:15, 7 cm from the nipple, was an elongated, irregular, hypoechoic mass measuring 1.4 × 0.6 × 1.4 cm, thought to represent a portion of the same entity. Given the finding of a new suspicious mass, an ultrasound-guided core biopsy was performed (Fig. 2). Pathology revealed an epidermal inclusion cyst (Fig. 3).

## Discussion

Epidermal inclusion cysts (EICs) result from proliferation and implantation of epidermal elements within a circumscribed space in the dermis [2]. They are the most common cutaneous or subcutaneous cysts and are lined by stratified squamous epithelium with a granular layer and lamellated keratin. They occur commonly in multiple areas of the body, including the trunk and extremities, and most frequently in the head and neck region. However, they are rarely found in the breast, with fewer than 40 cases reported [3]. They often present as a palpable breast lump and require work-up to differentiate them from other benign or malignant lesions of the breast. The present case provides clinicoradiological and cytohistological insight into EIC, especially in the setting of breast reduction mammoplasty.

EIC typically affects individuals in the fifth decade of life, both females and males. Various mechanisms that damage the epidermis and result in implantation of epidermal cells deep within the breast tissue may lead to EIC formation. Examples include congenital cysts secondary to obstructed hair follicles or pores, injury to the epidermis such as from needle biopsy or reduction mammoplasty in our case, or squamous metaplasia of normal columnar cells within a dilated duct [3,4]. During the reduction mammoplasty, the nipple/areola is repositioned along with the vascularized tissue pedicle, often by infolding of the tissue, which can cause the implantation of a fragment of the epidermis within the breast tissue, resulting in an epidermis cyst [5]. The diagnosis of an EIC in the subcutaneous tissue is straightforward; however, enlarged cysts located in the breast parenchyma present difficulty in differentiating them from other benign lesions such as fibroadenoma or phyllodes tumor, or from malignant breast lesions.

On physical examination, an EIC typically appears as a smooth, round nodule. On mammography, an EIC usually appears as a well-circumscribed mass that is isodense to hyperdense, typically without calcification, although older cysts may present with heterogeneous microcalcifications [[6], [7]]. On sonography, a breast EIC may appear as a solid, well-circumscribed, complex, or heterogeneous lesion. A specific sonographic feature—the “onion-ring” appearance—has been described [8]. On magnetic resonance imaging (MRI), an intermediate- to high-T2 signal mass with low-signal debris and without central enhancement has been reported as a helpful feature [9].

Potential complications of EICs include spontaneous rupture, infection, abscess formation and malignant transformation into squamous cell carcinoma which has been reported in 0.5%-19% of cases [10]. Malignant transformation occurs more frequently in the breast than in other parts of the body, and a significant correlation between tumor size and malignant transformation has been identified. Therefore, asymptomatic, small-sized lesions (< 2 cm) with typical sonographic findings do not require biopsy or treatment with close follow up to assess for stability [1]. However, large and palpable lesions causing physical or psychological discomfort may warrant surgical removal with wide elliptical excision for pathologic analysis and to prevent malignant transformation.

## Conclusion

Epidermal inclusion cysts are uncommon benign findings in the breast. Physical examination, ultrasound, and mammographic findings are nonspecific, making it radiographically difficult to exclude a malignant lesion. This case demonstrates that, in the setting of reduction mammoplasty, a new well-circumscribed mass on mammogram, with a sonographic appearance of an onion ring and an MR finding of an intermediate- to high-T2 signal mass with low debris and no central enhancement, should prompt consideration of an EIC.

## Patient consent

Written, informed consent for publication of this case, including relevant clinical details and any accompanying images, was obtained from the parent/legal guardian of the patient, who is a minor. The parent/legal guardian has reviewed the material to be published and has voluntarily agreed to its use for academic and scientific purposes. They understand that the patient’s identity will be protected and not revealed in the publication.
